# Bacterial diversity and community assembly mechanisms in alpine wetland soils under the influence of grazing

**DOI:** 10.1128/spectrum.03791-25

**Published:** 2026-07-06

**Authors:** Xu Ai, Cao Yu, Ji Yangyue, Zou Shuzhen, Kang Di

**Affiliations:** 1 Key Laboratory of Southwest China Wildlife Resources Conservation (Ministry of Education), China West Normal University 56714, Nanchong, China; 2 Laboratory of Environmental Science and Biodiversity Conservation, China West Normal University 56714, Nanchong, China; Commonwealth Scientific and Industrial Research Organisation, Brisbane, Australia

**Keywords:** alpine wetland, soil bacterial, community assembly, co-occurrence network, null model

## Abstract

Wetlands are among the world’s most significant ecosystems. Studying how different grazing intensities affect soil bacterial diversity and community assembly in alpine wetlands is crucial for their sustainable management. Based on 16S sequencing technology, this study analyzed the differences in the composition, structure, and community assembly mechanisms of bacterial communities under different grazing impacts on the Qinghai-Tibet Plateau. The research results showed that under the influence of grazing, there was no significant difference in the α-diversity of the soil bacterial community, but a certain degree of reorganization occurred at the phylum level of bacteria. At the same grazing intensity, the relative abundances of different bacterial phyla in the wetland were different, and at different grazing intensities, the relative abundances of the same phylum might have significant differences. Co-occurrence network analysis indicated that wetlands with medium grazing intensity had a higher degree of modularity. The results of the null model and neutral model showed that, with the increase in grazing intensity, the assembly process of the soil bacterial community gradually shifted from being dominated by deterministic processes to being dominated by neutral processes, with a decrease in heterogeneous selection and an increase in dispersal limitation.

**IMPORTANCE:**

This work innovatively clarifies grazing’s regulatory effects on alpine wetland soil bacterial community assembly, revealing the deterministic-to-neutral shift with increasing grazing intensity and higher modularity under moderate grazing. It deepens understanding of “disturbance-microbial assembly” relationships, bridging environmental science, disturbance ecology, and microbiology. Practically, it provides a scientific basis for optimizing grazing management to maintain microbial function, crucial for sustainable management of Qinghai-Tibet alpine wetlands and similar ecosystems.

## INTRODUCTION

Microorganisms are core components of wetland soil systems and serve as the basis for driving key ecological functions such as energy flow, nutrient transformation, and pollutant degradation (1). In the soil ecosystem, the number of bacteria often exceeds 90% of the total microbial population, far exceeding the proportion of fungi. Moreover, the composition and diversity of bacteria, as well as bacterial community assembly processes, are sensitive to environmental changes and can be used as important indicators for predicting the functional dissimilation of soil following environmental changes (2, 3). Alpine wetlands are among the most important natural ecosystems on Earth (4, 5), and their material cycles are regulated by bacterial activities (6, 7).

Grazing is a typical type of disturbance on alpine wetlands. It can alter the composition, structure, and function of soil microorganisms by changing soil properties (8, 9) and soil nutrients, thereby influencing the community assembly processes of wetland soil microorganisms. For example, livestock foraging on plants reduces the input of litter and root exudates for microorganisms and regulates the microbial community composition by changing the reserves of carbon sources and other material energy sources (10, 11). The hoof-tillage effect modifies the soil pore structure, leading to a decline in soil aeration and a decrease in the efficiency of oxygen penetration and diffusion (12–14). The input of livestock urine locally and instantaneously increases the soil pH and nitrogen content (15). These impacts increase environmental selection pressure, thus weakening the effects of processes such as dispersal limitation and ecological drift on community assembly and expanding the proportion of deterministic processes. Moreover, the microorganisms on the livestock body surface and in the intestinal tract spread to different areas during grazing activities, which to some extent enhances the influence of stochastic processes on community assembly (16). Therefore, the impact of grazing on microbial community assembly is complex.

The formation of local communities influenced by both environmental filtering and biological interactions is referred to as community assembly (17), which is determined by four fundamental processes: selection, drift, diversification, and dispersal (18). These four basic processes are typically classified into deterministic and stochastic processes. Owing to differences in habitat preferences and microbial adaptability, the assembly of microbial communities is affected by deterministic biotic and abiotic factors, which are known as deterministic processes (19–21). Conversely, stochastic processes such as birth, death, speciation, and limited dispersal shape community structure, which is a stochastic process (22, 23). The environmental changes caused by grazing can alter the relative contributions of stochastic and deterministic processes to community assembly. At the local level, stochastic processes dominate the assembly of bacterial communities, whereas at the regional level, the assembly of bacterial communities is dominated by deterministic processes (24, 25). Studies in alpine meadows have shown that grazing at different intensities reduces the role of homogeneous selection and increases dispersal limitation, resulting in a decrease in the contribution of deterministic processes to community assembly and an increase in the contribution of stochastic processes to community assembly (26). However, in fragile alpine wetlands, community assembly processes are complex and variable, and further research and verification are needed.

The Qinghai-Tibet Plateau, known as the “Third Pole of the Earth,” is one of the most representative alpine ecosystems globally. It is a key area for maintaining regional biodiversity and a crucial repository for supporting the sustainable development of plateau animal husbandry. Its ecological conditions profoundly impact the ecological security of China and even Asia (27). However, long-term overgrazing has led to significant degradation of the grassland ecosystem in this region. Problems such as a significant decline in vegetation and dominant forage coverage, an increase in soil bulk density, and exacerbated soil nutrient loss have emerged in many areas. The synergistic cycling mechanism of soil-plant bacteria has been disrupted (28, 29). Therefore, conducting research and ecological restoration in this area is urgent and necessary.

In this study, we focused on the diversity, community composition, and community assembly processes of wetland soil bacteria. We propose the following hypotheses: (i) different grazing intensities lead to changes in the composition and structure of wetland bacteria on the Qinghai-Tibet Plateau; (ii) grazing intensity can regulate the stochasticity of the community assembly process of alpine wetland soil bacteria. This study aims to explain how the soil bacterial communities in alpine wetlands adapt to the impacts of grazing and provide insights into predicting the responses of ecosystems to environmental changes. The conclusions of this study will contribute to scientifically predicting the risk of decline in ecosystem service functions caused by wetland grazing.

## MATERIALS AND METHODS

### Site selection

**Fig 1:**
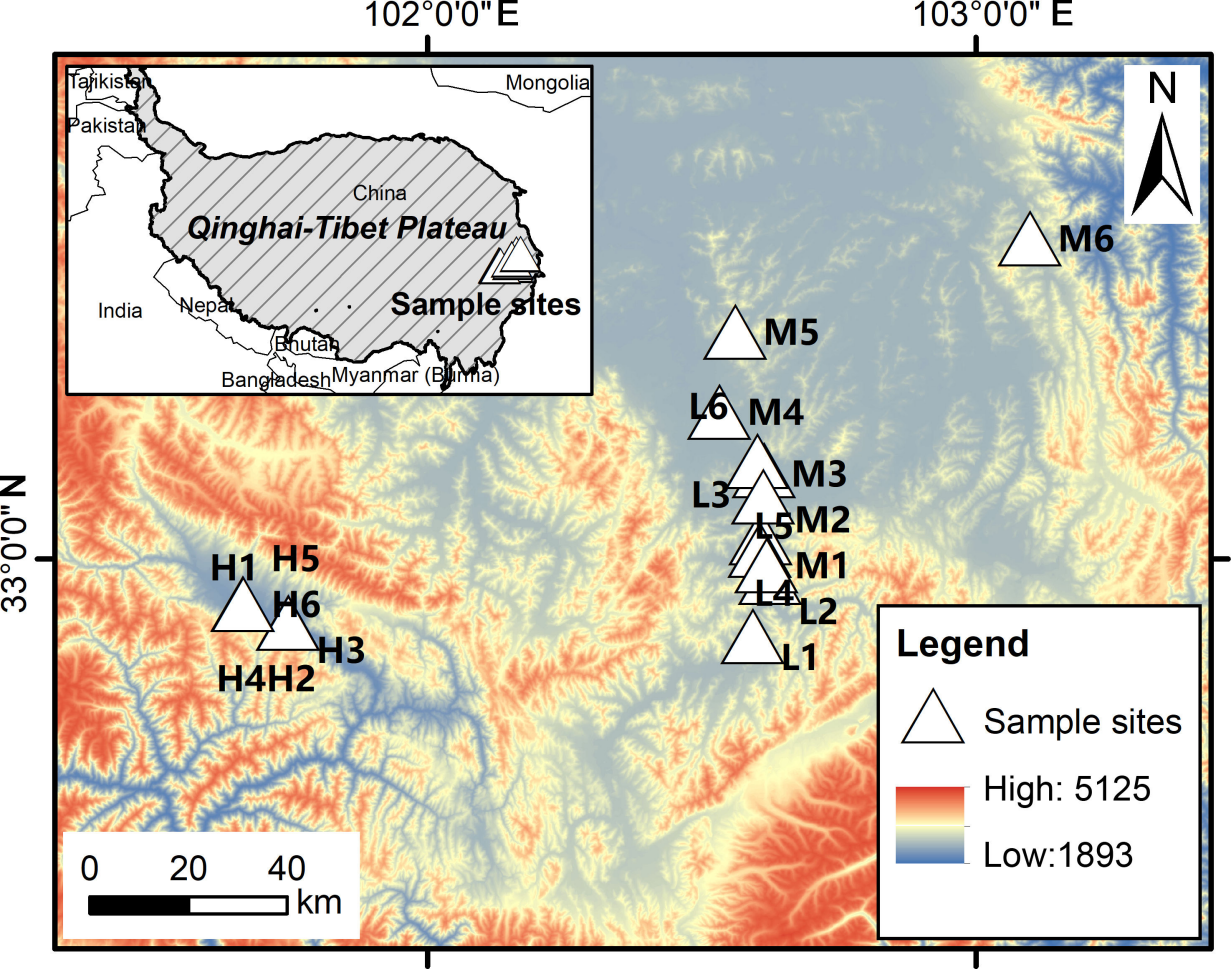
Location of sample sites. The map was created by the authors specifically for this study using ArcGIS 10.2 and publicly available geographic data sources.

The sampling area of this study, located at 33.50–32.50°N and 101.50–103.20°E, lies on the eastern fringe of the Qinghai-Tibet Plateau (Fig. 1). With an average altitude ranging from 3,400 m to 3,800 m, the region features a plateau-temperate semiarid monsoon climate. The annual average temperature is between 0.9°C and 2.5°C, and the average precipitation is 518 mm–800 mm. The growing season here is short, occurring mainly from July to September, whereas the nongrowing season extends for as long as 10 months. The alpine wetlands on the eastern margin of the Qinghai-Tibet Plateau represent the largest and most concentrated alpine peat swamp wetland areas in China. As a classic example of an alpine ecosystem, they have complete ecological components and an intact structure, serving as a vital water conservation zone for the upper reaches of the Yangtze and Yellow Rivers. The Baihe River, Suomo River, and Ake River flow through the sampling region, with average annual flow rates ranging from 50 m³/s–100 m³/s. The dominant plant species in this area include Carex alatauensis, Carex muliensis, other Carex-related species (if repeated Carex alatauensis is an error), Elymus nutans, Poa chalarantha, and Cremanthodium brunneopilosum.

### Field survey and sample collection

After investigation, 18 sampling areas were selected, covering six wetlands each with low grazing intensity (L), medium grazing intensity (M), and high grazing intensity (H). Their altitude (ALT), slope (SLO), and aspect (ASP) were measured (Fig. 1). On the basis of remote-sensing image analysis and site investigation of animal trampling traces, the scope of each wetland was determined according to the grazing intensity classification criteria proposed by scholars (30): low grazing intensity (normal grassland vegetation composition, with no or only a few animal trampling and fecal traces, and forage utilization rate ranging from 0% to 40%); medium grazing intensity (normal grassland vegetation composition, with animal trampling and fecal traces, forage utilization rate of 40% to 50%); and high grazing intensity (abnormal grassland vegetation composition, i.e., a significant decrease in forage coverage, a significant increase in weed coverage, widespread trampling and fecal traces, forage utilization rate >50%). In this area, a mixed livestock-raising method with a ratio of 6:4 in terms of the number of individuals of yak (Bos grunniens) and Tibetan sheep (Ovis aries) is adopted. The average age of yaks is 3.5 years, and they prefer to graze on the above-ground parts of sedges and rushes. The average age of Tibetan sheep is 1 year, and they prefer to graze on the leaves and stems of Stipa chinensis and *Poa pratensis*, complementing the foraging preferences of yaks. In each sampling area, a 20 m × 20 m quadrat was established, and three randomly selected 5 m × 5 m sampling points within the quadrat were used for soil sampling. When sampling, animal feces were avoided, and visible plant litter, fine roots, and animal residues were removed. Then, an appropriate amount of 0 cm–15 cm surface soil was collected via a soil auger with an inner diameter of 10 cm. The sample soils from each sampling point were mixed into a composite soil sample. One part was used for analyzing the basic soil properties, and the other part was stored at −80°C for genomic sequencing.

### Determination of soil chemical properties

The soil organic matter (SOM) content was determined via the external heating method with concentrated sulfuric acid-potassium dichromate. The total nitrogen (TN) in the soil was measured via the Kjeldahl method. The total phosphorus (TP) content was determined via the acid dissolution method. The total potassium (TK) content was measured via the sodium hydroxide alkali dissolution method. The Olsen method and flame photometry were employed to measure the available phosphorus (AP) and available potassium (AK) in the soil. The soil pH was evaluated via a pH monitor (Thermo Orion 868, MA, USA). The soil water content (WC) was determined via the drying method.

### Determination of soil microbial genomes and taxonomic assignment

Total community genomic DNA was extracted from soil samples via the E.Z.N.A. Soil DNA Kit (Omega BioTek, USA). The hypervariable region V3-V4 of the bacterial 16S rRNA gene was amplified with primer pairs 338F (5′-ACTCCTACGGGAGGCAGCAG-3′) and 806R (5′-GGACTACHVGGGTWTCTAAT-3′) by T100 Thermal Cycler PCR thermocycler (Bio-Rad, USA). The PCR reaction mixture included 4 μL 5 × Fast Pfu buffer, 2 μL 2.5 mM dNTPs, 0.8 μL each primer (5 μM), 0.4 μL Fast Pfu polymerase, 10 ng of template DNA, and ddH_2_O to a final volume of 20 µL. PCR amplification cycling conditions were as follows: initial denaturation at 95°C for 3 min, followed by 27 cycles of denaturation at 95°C for 30 s, annealing at 55°C for 30 s, and extension at 72°C for 45 s, and a single extension at 72°C for 10 min, ending at 4°C. Purified amplicons were pooled in equimolar amounts and paired-end sequenced on an Illumina NextSeq 2000 platform (Illumina, San Diego, USA) according to the standard protocols of Majorbio Bio-Pharm Technology Co. Ltd. (Shanghai, China). DADA2 denoised sequences are usually called amplicon sequence variants (ASVs). To minimize the effects of sequencing depth on α- and β-diversity measures, the number of sequences from each sample was rarefied to 20,000, which still yielded an average Good’s coverage of 97.90%. Taxonomic assignment of ASVs was performed using the Naive Bayes consensus taxonomy classifier implemented in QIIME2 and the SILVA 16S rRNA database (v.138).

### Statistical analysis methods and software

In this study, in accordance with the methods of Kang et al. (31), the Shannon index and Simpson index were used to quantify the α-diversity of the soil microbial communities. The differences in bacterial α-diversity and the abundances of major phyla under different grazing intensities were analyzed via *t*-tests. The β-diversity of the community was evaluated based on the relationships among the Bray-Curtis distance, environmental factors, altitude, and geographical distance. The altitude and geographical distance were measured in meters and kilometers, respectively. Before analysis, the data were normalized via the Z-score method. The calculations were completed via the vegan package (32) in R 4.3.3 and SPSS 21. One-way analysis of variance (ANOVA) was used for significance testing via SPSS 21, and the significance levels are marked with letters: values with a shared letter: *P* < 0.05; values with completely different letters: *P* < 0.01. Redundancy analysis (RDA, Canoco 5.0, USA) was adopted to study the correlations between specific environmental factors, bacterial diversity, and key bacterial taxa. Pearson analysis heatmaps and RDA plots were drawn in Origin 2021.

Phyla with a relative abundance greater than 0.01% were selected. To investigate the co-occurrence patterns, we first preprocessed the ASV abundance matrix by retaining only ASVs detected in at least 20% of samples to reduce false correlations induced by low-detection-rate taxa. The filtered ASV matrix was then aggregated to the phylum level, and phyla with a relative abundance greater than 0.01% across all samples were selected for subsequent co-occurrence analysis. Pairwise correlations among the selected phyla were calculated based on the aggregated phylum-level abundance matrix. If the Spearman correlation coefficient was greater than 0.6 and *P* < 0.05, the co-occurrence pattern was considered stable. Gephi was used to calculate and visualize the number and degree of nodes and edges. In the network-node analysis diagram, nodes represent individual bacterial genera, and edges represent pairwise relationships within the bacterial community network, indicating significant biological or biochemical interactions between bacterial communities. The top 10% of nodes in terms of network degree and closeness centrality were regarded as hub taxa. To quantify the contributions of key ecological processes to the assembly of bacterial communities, the βNTI (33) combined with the Raup-Crick method of Bray-Curtis distances was adopted. Moreover, the Sloan neutral community model was used to infer the contributions of stochastic processes. The fitness *R*² was used to infer stochastic processes, and “m” was used to represent the migration rate. A higher “m” value indicates more significant dispersal limitation. The results were visualized via the ggplot2 package in R 4.3.3 and Origin 9.8 (OriginLab Corporation, 2021).

## RESULTS

### α-Diversity and community composition of wetland bacteria under the influence of grazing

**Fig 2:**
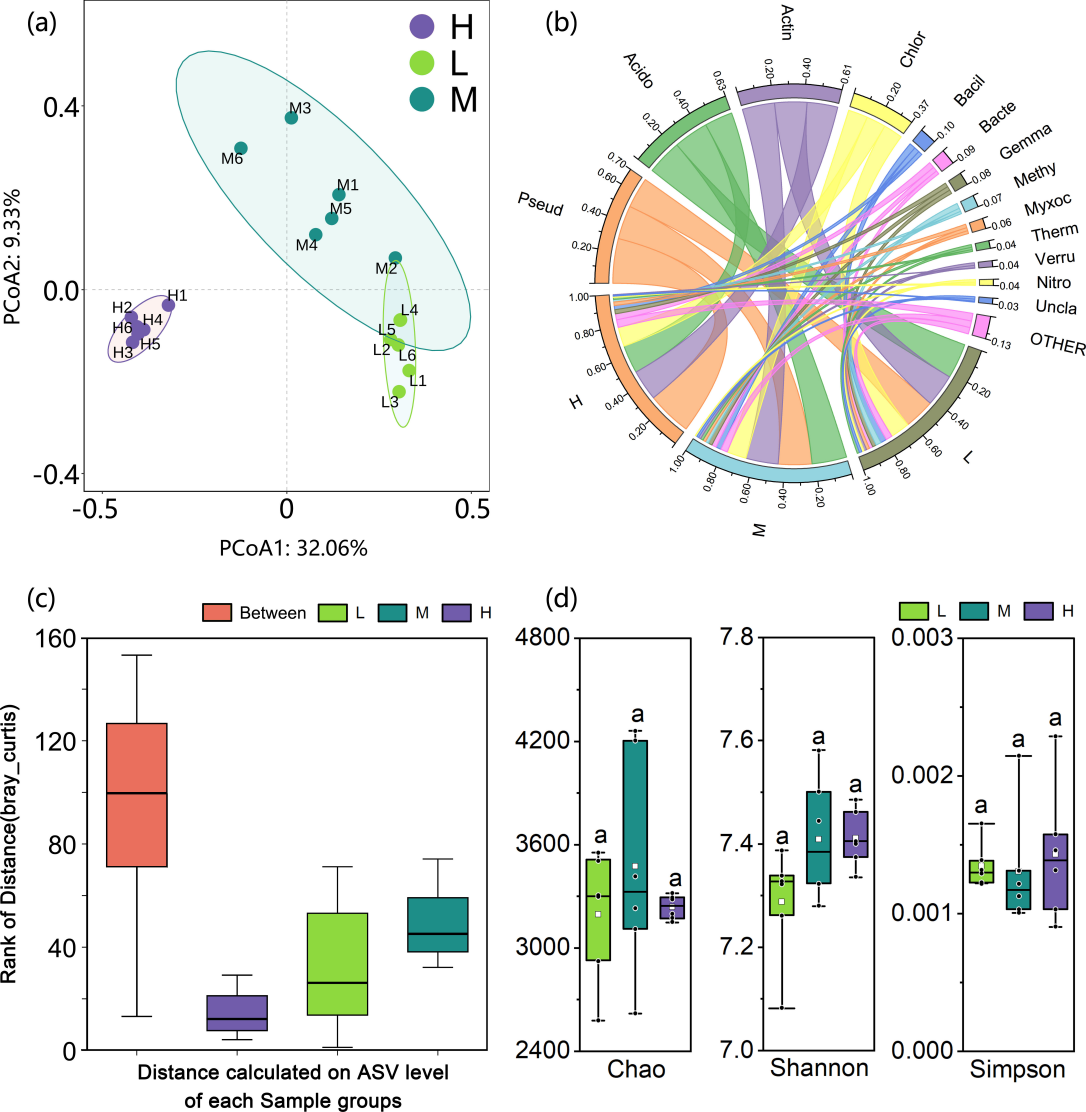
α-Diversity and community composition of soil bacteria under grazing conditions. (**a**) PCoA of clustering of bacterial composition. (**b**) Differences in the abundances of major bacterial phyla (relative abundance > 0.01%) under different grazing intensities. (**c**) ANOSIM analysis of differences in species composition. (**d**) α-Diversity. Abbreviations: L represents low grazing intensity; M represents medium grazing intensity; H represents high grazing intensity; between represents different grazing intensities. In panel **a**, the confidence interval is 95%; in panel **b**, the arc of the outer ring represents the relative abundance of the bacterial phylum. The larger the arc is, the greater the relative abundance of the bacterial phylum; in panel **d**, “a” represents the result of one-way ANOVA, indicating that there is no significant difference in the α-diversity index among the different grazing intensities.

Principal coordinate analysis (PCoA) and analysis of similarities (ANOSIM) were conducted on the microbial compositions at all the sampling points. The results revealed that the bacterial compositions clustered on the basis of differences in grazing intensity (Fig. 2a), and the differences between groups were greater than those within groups (Fig. 2c). The distribution range of the bacterial community in wetlands with high grazing intensity was narrower, with a lower degree of dispersion. Its confidence interval was separated from and had a large distance to the 95% confidence intervals of wetlands with low and medium grazing intensities, indicating a significant difference. The 95% confidence intervals of wetlands with low and medium grazing intensities were somewhat separated to a certain extent but still had some overlap.

The bacterial diversity indices in alpine wetlands under low, medium, and high grazing intensity disturbances were studied (Fig. 2d). The results showed that the species richness, diversity, and dominance in wetlands under medium grazing intensity were at a relatively high level, followed by low grazing intensity, and then high grazing intensity. There was no significant difference in the α-diversity of soil microorganisms. A further examination of 14 phyla with relatively high abundances (relative abundance >0.01%) (Fig. 2b; Table 1) revealed that the relative abundances of bacterial phyla in wetlands varied under different grazing intensities. For the same phylum, significant differences in relative abundance might exist among different grazing intensities. Specifically, between wetlands with low–medium grazing intensities, only the relative abundance of Methylomirabilota was extremely significantly different, whereas that of Bacteroidota was significantly different. The relative abundances of Bacteroidota and Verrucomicrobiota significantly differed between wetlands with low and high grazing intensities, whereas those of Bacteroidota, Gemmatimonadota, and Methylomirabilota, as well as unclassified bacteria and other phyla, significantly differed. The relative abundances of Bacteroidota, Myxococcota, Verrucomicrobiota, unclassified bacteria, and other phyla significantly differed between wetlands with medium and high grazing intensities.

**TABLE 1:** Characterization and difference of soil bacterial phyla composition in alpine wetlands under different grazing intensities^*a*^

Phylum	L	SD_L_	M	SD_M_	H	SD_H_
Pseudomonadota	0.197 a	0.016	0.216 a	0.026	0.287 b	0.054
Acidobacteriota	0.226	0.031	0.223	0.035	0.181	0.075
Actinomycetota	0.207	0.039	0.208	0.03	0.197	0.016
Chloroflexota	0.124	0.024	0.125	0.019	0.125	0.01
Bacillota	0.04	0.012	0.034	0.031	0.026	0.013
Bacteroidota	0.023 a	0.006	0.029 ab	0.011	0.039 b	0.017
Gemmatimonadota	0.024	0.005	0.026	0.003	0.027	0.007
Methylomirabilota	0.034 b	0.015	0.021 ab	0.011	0.016 a	0.005
Myxococcota	0.02 ab	0.003	0.018 a	0.003	0.023 b	0.006
Thermodesulfobacteriota	0.015	0.006	0.017	0.007	0.012	0.008
Verrucomicrobiota	0.017 b	0.007	0.015 b	0.009	0.003 a	0.001
Nitrospirota	0.016	0.009	0.011	0.003	0.009	0.006
Unclassified bacteria	0.015 b	0.006	0.013 b	0.007	0.006 a	0.002
Other	0.041	0.005	0.043	0.012	0.049	0.005

^*a*^ SD means standard error, the values indicate the relative abundance of each bacterial phylum within the community. Values followed by the same letter are not significantly different (*P* > 0.05), while values sharing one letter indicate significant difference between samples at *P* < 0.05, and values with completely different letters indicate highly significant difference between samples at *P* < 0.01.

We explored the relationships between the βNTI of alpine wetland bacteria and environmental factors (Fig. 5c). The results revealed that the pH of alpine wetlands had an extremely significant effect (*P* < 0.01) on the β-diversity of bacteria, with a positive correlation. Environmental factors such as ALT (*P* < 0.01), CD (*P* < 0.01), TN (*P* < 0.01), SOM (*P* < 0.01), and TP (*P* < 0.01) had extremely significant impacts on the β-diversity of bacteria, with a negative correlation. The β-diversity of bacteria was significantly affected by SLO (*P* < 0.05) and AK (*P* < 0.05), whereas ASP, AP, and WC had no significant effect on the β-diversity of bacteria.

**Fig 3:**
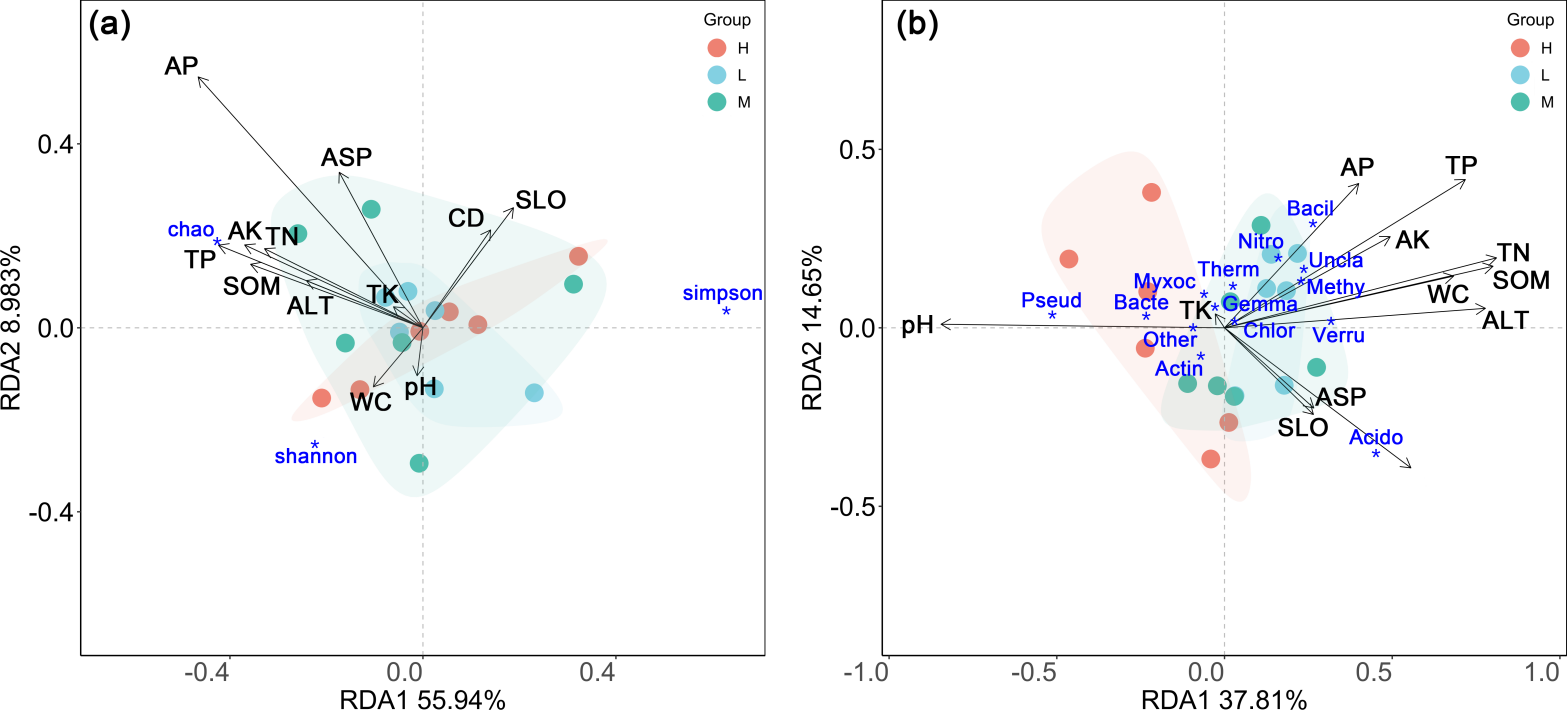
RDA analysis between bacterial diversity and environmental factors. (**a**) RDA analysis between bacterial diversity and environmental factors. (**b**) RDA analysis between key bacterial taxa and environmental factors. ALT: altitude; SLO: slope; ASP: aspect; CD: plant coverage; TN: total nitrogen in soil; SOM: soil organic matter; TP: total phosphorus in soil; TK: total potassium in soil; AK: available potassium in soil; AP: available phosphorus in soil; pH: soil pH; WC: soil water content; the dots L, M, and H represent sampling points; the arrows of variable loadings represent environmental factors.

RDA and correlation analysis were used to explore the correlations between bacterial diversity and environmental factors (Fig. 3a; Table S2), aiming to characterize the interactions between the microenvironments formed under different grazing intensities and bacterial diversity. RDA1 accounted for 55.94% of the total variance, and RDA2 accounted for 8.983% of the total variance, with a combined total of 64.923%. The results revealed that the Chao diversity index was positively correlated with ALT, SOM, TN, AK, TP, AP (*P* < 0.01), and ASP and negatively correlated with CD and SLO. The Shannon index was positively correlated with ASP, AK, AP, and pH and negatively correlated with ALT, SLO, CD, TN, SOM, TP, TK, and WC. The Simpson index was positively correlated with SLO, CD, and pH and negatively correlated with ALT, ASP, TN, SOM, TP, TK, AK, AP, and WC.

RDA and Pearson correlation analysis were used to explore the associations between key bacterial taxa and environmental factors (Fig. 3b; Table S3), aiming to characterize the interactions between the microenvironments formed under different grazing intensities and key bacterial taxa. RDA1 accounted for 37.81% of the total variance, and RDA2 accounted for 14.65% of the total variance, with a combined total of 52.46%. The results revealed that Pseudomonadota was extremely significantly negatively correlated (*P* < 0.01) with ALT, TN, SOM, TP, and WC; significantly negatively correlated (*P* < 0.1) with CD; and extremely significantly positively correlated (*P* < 0.01) with pH. Acidobacteriota had a significant positive correlation (*P* < 0.1) with CD and a significant negative correlation (*P* < 0.1) with pH. Actinomycetota had a significant positive correlation (*P* < 0.1) with SLO. Bacillota had an extremely significant positive correlation (*P* < 0.01) with TP and AK and a significant positive correlation (*P* < 0.1) with TN, SOM, TP, and AP. The abundance of Bacteroidota was significantly positively correlated (*P* < 0.1) with pH and significantly negatively correlated (*P* < 0.1) with CD. Methylomirabilota had an extremely significant positive correlation (*P* < 0.01) with WC, a significant positive correlation (*P* < 0.1) with TP, and a significant negative correlation (*P* < 0.1) with pH. The Verrucomicrobiota was extremely significantly positively correlated (*P* < 0.01) with ALT, TN, SOM, TP, and AK; significantly positively correlated (*P* < 0.1) with WC; and extremely significantly negatively correlated (*P* < 0.01) with pH. NB1-j had extremely significant positive correlations (*P* < 0.01) with pH and extremely significant negative correlations (*P* < 0.01) with ALT, CD, TN, SOM, and TP; and significant negative correlations (*P* < 0.1) with pH. Cyanobacteria had an extremely significant positive correlation (*P* < 0.01) with pH; extremely significant negative correlations (*P* < 0.01) with ALT, TN, and SOM; and significant negative correlations (*P* < 0.1) with CD, TP, and AP. Armatimonadetes had an extremely significant positive correlation (*P* < 0.01) with pH and a significant negative correlation (*P* < 0.1) with ALT, TN, and SOM. In summary, grazing disturbance had no significant effect on the diversity of soil bacteria in alpine wetlands, but to some extent, it led to significant changes in their composition at the phylum level.

### Patterns of changes in the symbiotic networks of wetland soil bacteria under the influence of grazing

**Fig 4:**
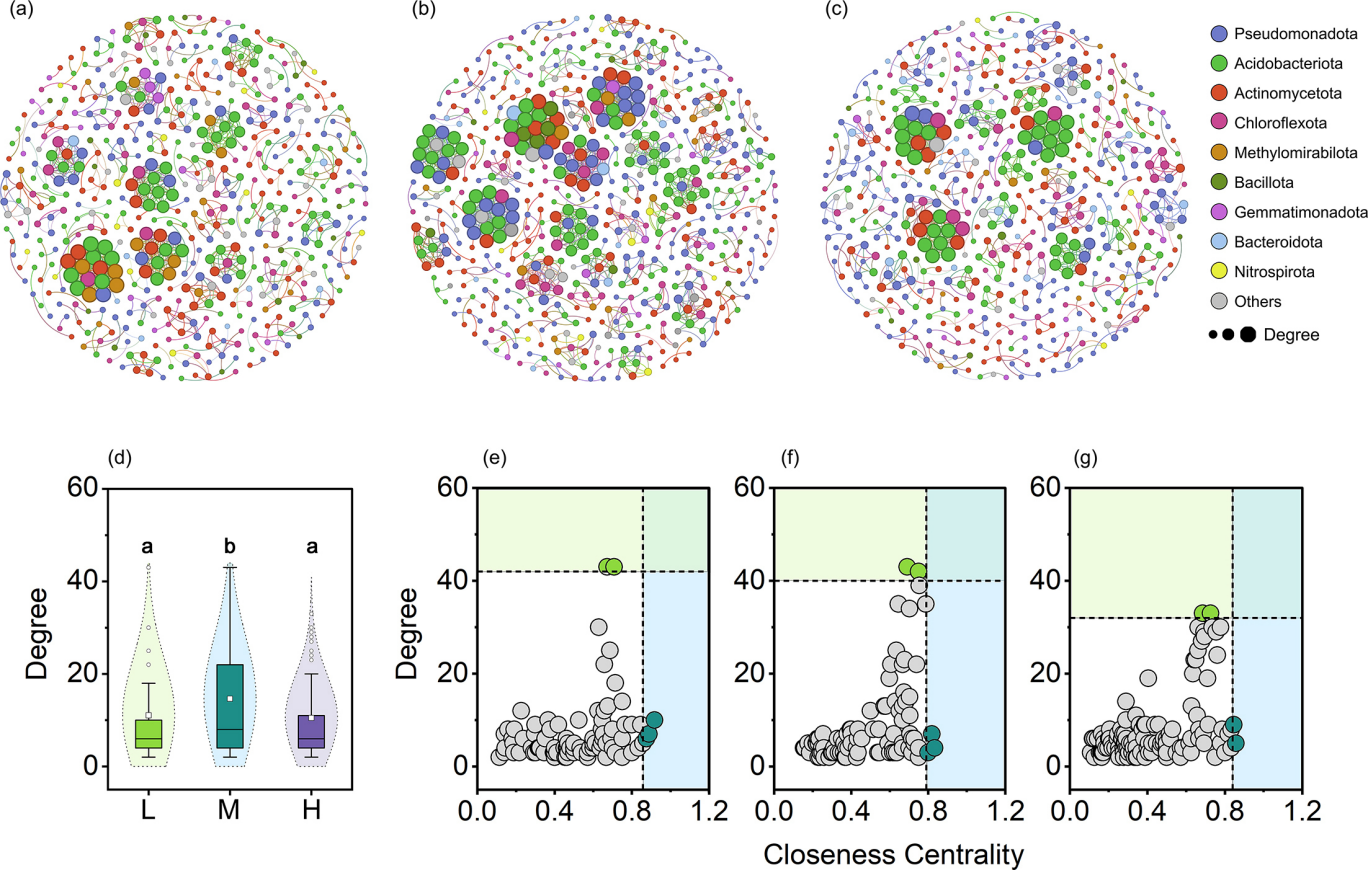
Co-occurrence network analysis. (**a**) Co-occurrence network of soil bacteria in wetlands with low grazing intensity. (**b**) Co-occurrence network of soil bacteria in wetlands with medium grazing intensity. (**c**) Co-occurrence network of soil bacteria in wetlands with high grazing intensity. In the co-occurrence networks, circles represent nodes. The color of the circle represents different bacterial phyla, and the size of the circle represents the high or low degree value of the node. The connecting lines represent edges, and the darker the color of the edge, the higher its weight. (**d**) Topological characteristic parameters at the node level; the *P* value represents the result of one-way ANOVA, indicating the degree of difference in network topology among grazing intensities. (**e**) Analysis of the hub taxa of bacteria in low-grazing-intensity wetlands. (**f**) Analysis of the hub taxa of bacteria in medium-grazing wetlands. (**g**) Analysis of the hub taxa of bacteria in high-grazing-intensity wetlands.

Under three grazing intensities (low, medium, and high), ASVs with a relative abundance greater than 0.0002% were selected to construct symbiotic networks for all bacteria, and the topological characteristics of the network nodes and edges were calculated. The results of the network analysis revealed that the wetlands with low grazing intensity had 530 nodes and 912 edges (Fig. 4a), whereas the wetlands with medium grazing intensity had 580 nodes and 1,363 edges (Fig. 4b), and the wetlands with high grazing intensity had 580 nodes and 784 edges. For all bacteria involved in the study, the average degree of wetlands with low grazing intensity was 3.442, the average degree of wetlands with medium grazing intensity was 4.7, and the average degree of wetlands with high grazing intensity was 2.953. Based on the above results, we used degree to characterize the topological features of the network (Fig. 4d). The results indicated that the degree of wetlands with medium grazing intensity was significantly greater than that of low- and high-grazing-intensity wetlands (*P* < 0.01). Under this grazing intensity, the bacterial network structure was the most intensive, and the interactions between taxa were the strongest. There was no significant difference in degree between low- and high-grazing-intensity wetlands, and both were significantly lower than that of medium-grazing-intensity wetlands. This finding indicates that the bacterial network structure in low-grazing-intensity wetlands was relatively loose, with weak interactions between taxa, whereas the bacterial network structure in high-grazing-intensity wetlands was the loosest, with the weakest interactions between taxa.

We defined the taxa ranked in the top 10% in terms of degree and closeness centrality as hub taxa. The results revealed that there were 44 bacterial taxa in the soil of wetlands with low grazing intensity, whereas the number of bacterial taxa in the soil of wetlands with medium and high grazing intensities decreased to 37 and 36, respectively. There were no top-level hub taxonomic units under low, medium, or high grazing intensities. However, there were five nodes close to centrality in the wetlands with low and medium grazing intensities, whereas the number of nodes close to centrality decreased to four in the wetlands with high grazing intensity.

### Patterns of bacterial community assembly under the influence of grazing

**Fig 5:**
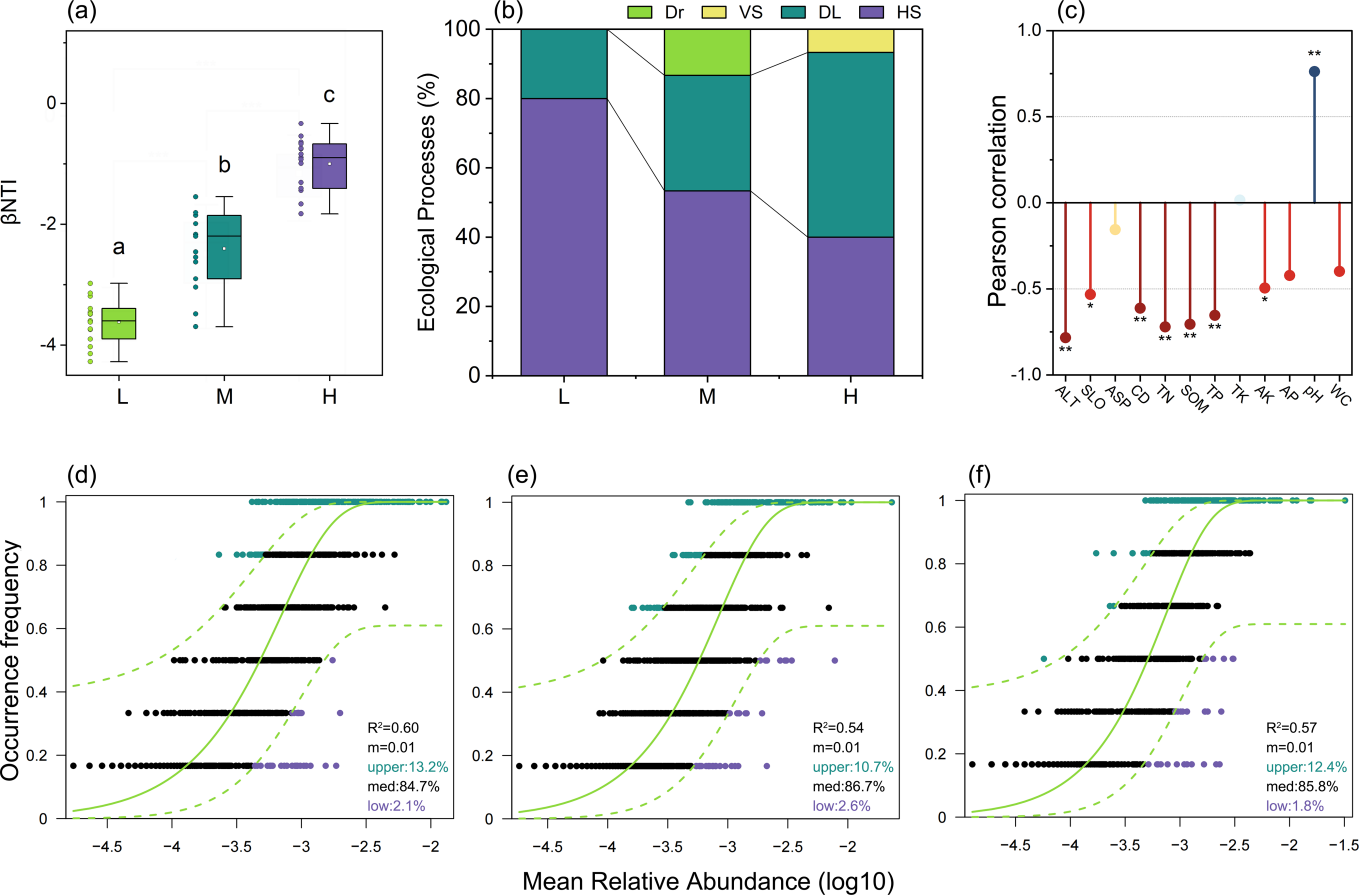
Assembly processes of wetland soil bacterial communities. (**a**) Different letters following the values indicate significant differences between samples at *P* < 0.05. (**b**) Contributions of different ecological processes to the assembly of bacterial communities. (**c**) Correlation analysis between βNTI and environmental factors. (**d–f**) Neutral community model of L, M and H. In the neutral model, the green and purple circles represent species with occurrence rates higher or lower than those predicted by the model. The solid green line represents the best fit to the neutral community model, and the dashed green line represents the 95% confidence interval. “m” represents the estimated migration rate, and “R2” represents the goodness-of-fit to the neutral community model. *, *P* < 0.05; **, *P* < 0.01; ***, *P* < 0.001.

In this study, the null model and Sloan neutral model were employed to investigate the assembly processes of soil bacterial communities along environmental gradients. Based on the results of the null model, we hypothesized that deterministic processes (|βNTI| > 2) dominated the assembly process of the bacterial community in wetlands with low grazing intensity. For wetlands with medium grazing intensity, although deterministic processes partially dominated community assembly, βNTI increased significantly (*P* < 0.001), indicating that the community assembly at some sampling points had shifted to stochastic processes. The assembly process of the bacterial community in wetlands with high grazing intensity was dominated by stochastic processes (|βNTI| < 2) (Fig. 5a). Further analysis via the RCbray null model revealed the proportional dominance of different community assembly driving factors within the sampling points (Fig. 5b). The results revealed that as the grazing intensity increased, the proportion of homogeneous selection in the community assembly process of alpine wetland bacteria decreased, whereas the proportion of dispersal limitation increased. Ecological drift only played a role in wetlands with medium grazing intensity, and variable selection affected only wetlands with high grazing intensity. We used the migration rate (m) estimated by the Sloan neutral model to reflect the dispersal ability of the species (Fig. 5d through f) The migration rates of wetlands with low, medium, and high grazing intensities were all relatively low.

## DISCUSSION

At present, grazing is the most common way to utilize natural grasses. Grazing indirectly affects soil microbial communities by altering the microhabitat. Owing to their extreme and fragile environmental substrate, alpine wetlands are significantly more affected by disturbances such as grazing and global climate change than other wetland types are. Therefore, studying the impacts of grazing on alpine wetlands can provide scientific references for wetland ecological protection and restoration in more regions. Microorganisms play crucial roles in matter cycling and energy flow, and bacteria usually account for more than 90% of the total microbial quantity (34). Although some studies have explored soil microorganisms in alpine wetlands, the composition of soil microorganisms and the mechanisms of community assembly under grazing disturbance remain unclear. Thus, this study aims to explore these aspects.

### α-Diversity and community composition of wetland bacteria under the influence of grazing

The diversity of bacterial communities is crucial for assessing the state of ecosystems and uncovering their functions. However, owing to the environmental heterogeneity of different ecosystems, the patterns of bacterial diversity change. In this study, we found that there was no significant difference in the α-diversity of the soil bacterial communities in alpine wetlands under different grazing intensities. A previous meta-analysis supported these results. They noted that grazing significantly altered the structure of only soil bacterial communities and decreased β-diversity, whereas α-diversity remained stable (35). The core reason is that the broad-spectrum physiological adaptability of soil bacteria can maintain their species richness (26). However, Yao et al.’s study (36) on the Inner Mongolia grassland reached a different conclusion: with increasing grazing intensity, soil bacterial diversity decreased significantly (37). The difference may stem from the environmental differences between the study areas. Inner Mongolia is an arid and semiarid region. Grazing, through livestock trampling and vegetation degradation, exacerbates soil water loss, and water scarcity directly limits the survival of microorganisms, which is in sharp contrast to the relatively humid habitat of alpine wetlands. In summary, the impact of grazing on the α-diversity of soil bacteria is ecosystem-specific, and specific research in different ecosystems is needed.

To further clarify the impact of grazing on the bacterial community in alpine wetlands, this study analyzed the changes in 14 dominant bacterial phyla (relative abundance >0.01%). Under medium grazing intensity, wetlands showed high levels of species richness, diversity, and dominance. The levels were lower under low grazing intensity, and even lower under high grazing intensity. However, there was no significant difference in the α-diversity of soil microorganisms. At the phylum level, Methylomirabilota, Bacteroidota, Pseudomonadota, and Verrucomicrobiota are more sensitive to the environmental changes caused by differences in grazing intensity. Under intense grazing, the soil aeration decreases due to trampling, providing a suitable habitat for anaerobic Methylomirabilota (38). Bacteroidota can use cellulose, hemicellulose, pectin, and even plant root exudates as carbon sources. Grazing regulates the abundance of Bacteroidota by altering the input of these plant-derived carbons (39). Additionally, Pseudomonadota have high metabolic diversity (40). If their environment is disturbed, Pseudomonadota can utilize a variety of different substances as carbon and energy sources (41), so the relative abundance of Pseudomonadota is significantly higher under high grazing intensities (42). Meanwhile, Verrucomicrobiota is sensitive to environmental changes, and its abundance fluctuations are synchronized with the microenvironmental changes caused by grazing. Grazing leads to changes in the soil microenvironment, and different phyla have different sensitivity thresholds and responses to environmental factors, resulting in differences in the relative abundances of various phyla under different grazing intensities.

The results of this study indicate that grazing is an important factor influencing the assembly of soil bacterial communities in alpine wetlands. Changes in environmental factors caused by grazing affect the process of bacterial community assembly. An exploration of the relationships between the βNTI of alpine wetland bacteria and environmental factors revealed that the pH of alpine wetlands has an extremely significant and positive impact on the β-diversity of bacteria. The pH affects the survival and reproduction of soil bacteria by altering their metabolic pathways, enzyme activities, and availability and solubility of soil nutrients (43). Bacteria have a specific suitable pH range for survival. When the environmental pH approaches this range, the environmental selection pressure weakens, and the influence of stochastic processes on the assembly of wetland soil bacterial communities increases (44). This finding is consistent with the conclusion drawn by Kerfahi et al. from comparing bacterial communities in soils with extreme pH values, where stochastic processes dominate the bacterial communities in habitats with medium and high pH values. Environmental factors such as ALT, CD, TN, SOM, and TP have extremely significant and negative impacts on bacterial βNTI. This occurs because as the ALT increases, the temperature decreases, and the partial pressure of oxygen changes (45). When the CD changes, factors such as light, wetland conditions, and litter input also change. When the nutrient concentrations of TN, SOM, and TP in the soil change, if bacteria face a situation of nitrogen source deficiency (46) or phosphorus source shortage, their metabolism increases through mutations in related pathway genes (47). The environmental selection of bacteria is significantly increased, and deterministic processes dominate. The βNTI of the bacterial community was significantly and negatively affected by SLO and AK. An increase in SLO exacerbates soil erosion and differences in nutrient distribution (48), easily leading to the formation of heterogeneous resources and the dominance of deterministic processes in community assembly. When the AK concentration increases, high-concentration potassium can cause osmotic stress to some bacteria, affecting their growth rate and interspecies competition by directly participating in metabolic processes, thus changing the community structure and enhancing the influence of deterministic processes on the community assembly process. This finding is consistent with the conclusions of Hu et al. concerning the convergence and response of bacterial communities to salinization (49).

By means of RDA and correlation analysis, the influence of environmental factors on bacterial diversity was further clarified. The results revealed that surface environmental characteristics and soil quality were significantly correlated with bacterial community diversity. For example, ALT affects the formation of a local microclimate by increasing soil moisture (37, 50), and SOM and other nutrients provide abundant carbon and nitrogen sources, which is an important mechanism for maintaining bacterial diversity. This finding is consistent with the conclusion obtained by Wu et al. in their study on the bacterial community structure during soil acidification; that is, extreme pH values inhibit bacterial diversity, whereas changing the pH within a moderate range is conducive to increasing bacterial richness (51). The bacterial diversity index was negatively correlated with CD and SLO. An increase in CD reduces surface light, soil temperature, and wetland water level, which may limit the survival of some bacteria. An increase in SLO accelerates water evaporation and reduces the water-holding capacity (52). In addition, the Shannon index was negatively correlated with nutrients such as TN and SOM, as was WC. Excessive nutrients or water can lead to the overreproduction of dominant bacteria, inhibiting other bacterial groups and reducing evenness (53). The positive and negative correlation mechanisms of the Simpson index are consistent with the former two, so they will not be elaborated further. In summary, the correlations between bacterial diversity indices and various environmental factors vary, and different factors can promote or inhibit bacterial diversity by affecting the survival and metabolism of bacteria.

Moreover, the results of the RDA and correlation analysis indicate that the relationships between key bacterial taxa and environmental factors can be classified into three categories. The first is the pH-driven type. The abundances of Pseudomonadota, Cyanobacteria, and Armatimonadetes were significantly positively correlated with pH. This may be because in soils with high pH values, the availability of soil nutrients and the activity of the intracellular enzyme system are relatively high (54, 55), and the soil has a strong buffering capacity with a small range of acid-base changes, which is conducive to the growth of bacterial taxa sensitive to pH. Conversely, Acidobacteriota are acidophilic bacteria, Methylomirabilota rely on an acidic environment for the redox of methane (56), and Verrucomicrobiota have a greater ability to survive in low-pH environments (57), so their relative abundances are significantly negatively correlated with pH. The second is the nutrient-driven type. Bacillota and Verrucomicrobiota were significantly positively correlated with TP, AK, TN, and SOM. Bacillota can participate in the nitrogen cycle and release soil phosphorus and potassium to increase nutrient levels (58, 59). Verrucomicrobia can decompose organic matter and increase soil nutrients. The third is the water-driven type. Methylomirabilota were extremely significantly positively correlated with WC. This occurred because Methylomirabilota are anaerobic bacteria, and the anaerobic or hypoxic environment formed by high soil water content is conducive to their growth and metabolism. In contrast, the relative abundance of the aerobic bacterium Pseudomonadota was extremely significantly negatively correlated with WC. Different bacterial taxa have different biological characteristics, thus resulting in differences in their correlations with various environmental factors.

### Patterns of changes in the symbiotic networks of wetland soil bacteria under the influence of grazing

Our research results indicate that the modularity of the bacterial community in wetlands with medium grazing intensity is the highest, followed by that in wetlands with low grazing intensity, and the modularity is the lowest in wetlands with high grazing intensity. Similarly, Xiang et al. reported that grazing at an appropriate intensity creates a heterogeneous habitat for the bacterial community, promoting the modularization of the bacterial community (60). In contrast, high-intensity grazing may lead to habitat homogenization and a reduction in the modularity of the bacterial community (61). Under medium grazing intensity, the soil environment is rich in resources, and the environmental pressure is moderate, providing suitable living conditions for the bacterial community. This enables bacteria to form different functional modules through niche differentiation and maintain the stability and stress resistance of the community through the synergy between modules (62). The synergy within the soil community under grazing stress is a survival strategy of the soil microbial community (63). Under low grazing intensity, although the modularity is relatively high, the community complexity and stability are low. Under high grazing intensity, due to the high environmental pressure on the bacterial community, intense grazing activities may cause severe damage to the wetland soil ecosystem, deteriorate the living environment of the bacterial community, damage the synergy among the bacterial community, and thus reduce modularity and community stability (64). Therefore, moderate grazing has an optimizing effect on the structure and function of the bacterial community, whereas excessive grazing has a negative effect.

In addition, our research also revealed that, compared with those in wetlands with low and high grazing intensities, the soil bacteria in wetlands with medium grazing intensities presented shorter average paths and greater degrees of co-occurrence. Therefore, the network structure of soil bacteria in wetlands with medium grazing intensity is more compact and complex, with a higher co-occurrence frequency of bacterial taxa, whereas the bacterial community in wetlands with high grazing intensity has the opposite characteristics. Existing studies indicate that the co-occurrence network structure can reflect the complex interaction relationships among bacteria. There may be various ecological relationships, such as cooperation, coexistence, predation, and competition between connected nodes (65). Currently, regarding this phenomenon, two hypotheses have been proposed in the academic community. At present, existing research has formulated two main hypotheses for this phenomenon. One hypothesis suggests that a spatially compact bacterial community has greater connectivity, enhancing its interactions (66), to compensate for environmental pressure and thus maintain or improve community stability (67). The other hypothesis posits that increased environmental pressure or diversity may lead to a more compact network structure of the bacterial community (68). The characteristics of the network structure and community in this study are more consistent with the latter hypothesis. Although there is currently no exact method to accurately quantify the proportion of influence of various hypotheses on the bacterial community, the stress-gradient hypothesis indicates that an increase in environmental stress will lead to an increase in the proportion of positive interactions among organisms and a weakening of competition, thus promoting the tightening of the co-occurrence network structure (69). Meanwhile, an increase in diversity can strengthen species associations through niche differentiation and functional complementarity, further promoting the compactness of the co-occurrence network structure. In this study, the species richness, diversity, and dominance of the soil bacterial community under medium grazing intensity were at a relatively high level, and the degree of environmental stress was relatively high. Therefore, the characteristics of the network structure and the community characteristics are more consistent with the latter hypothesis.

Previous studies have shown that hub taxa play crucial roles in bacterial communities, driving community composition and function (70). The absence of hub taxa can simplify the network structure, alter the interaction patterns among microorganisms, and reduce the stability and functionality of the community (71, 72). Our research results indicate that, with increasing grazing intensity, the number of hub taxa of soil bacteria in alpine wetlands gradually decreases. This may be caused by changes in community composition and species loss, and it also implies that grazing has certain negative impacts on community stability (62).

### Patterns of bacterial community assembly under the influence of grazing

Through the study of the community assembly process of soil bacterial communities along the environmental gradient, it was found that as the grazing intensity increased, the community assembly process of bacterial communities gradually shifted from ecological niche process to neutral process. Although the null model and the neutral model have different perspectives on quantifying the neutral-ecological niche processes, essentially, both reveal the mechanism by which grazing intensity regulates the balance between neutral and ecological niche processes by changing the degree of disturbance. Further analysis of the community’s RCbray showed that, according to the null model, as the grazing intensity increased, the homogeneous selection process decreased, and the dispersal limitation process increased, which is consistent with the research conclusion of Que et al. on the impact of grazing intensity on the spatial heterogeneity of the constructive species in desert steppes (73). With the intensification of grazing, the foraging and trampling of wetland resources by organisms increased, significantly changing the soil physical and chemical properties within a small-scale habitat (74). The habitats of soil bacteria are damaged and even fragmented (75), thus weakening the impact of homogeneous selection and enhancing the impact of dispersal limitation. Meanwhile, as the grazing intensity increased, the goodness-of-fit of the neutral model (*R*^2^) first decreased and then slightly rebounded, which also confirmed the conclusion of the null model that as the grazing intensity increased, community assembly shifted from being mainly dominated by ecological niche processes to being mainly dominated by neutral processes.

Under low grazing intensity, due to minimal grazing disturbance, the ecosystem is relatively stable, and the life activities of microorganisms are less affected by external disturbances. Therefore, the contribution of the neutral process is the highest at this time (76). We also observed that ecological drift only affects wetlands with medium grazing intensity, and the goodness-of-fit *R*^2^ is slightly lower than that under low grazing intensity. This is because when both homogeneous selection and dispersal selection are moderate, although the environmental physical and chemical properties change, a certain degree of stability and connectivity is still maintained. Random events caused by microbial individuals are sufficient to affect the community assembly process of the soil microbial community, that is, ecological drift can affect the community composition. In addition, as the grazing intensity increases, factors such as environmental selection pressure and interspecies competition become stronger (77), reducing the explanatory power of neutral processes. Heterogeneous selection only affects wetlands with high grazing intensity, and the goodness-of-fit *R*^2^ slightly rebounds compared to that under medium grazing intensity. This may be significantly related to the fact that high-intensity grazing damages the wetland habitat, leading to the simplification of the wetland bacterial community structure (78), as well as the significant heterogeneity of carbon content, nitrogen content, plant richness, and soil electrical conductivity in wetland soils (79, 80), causing changes in the direction or intensity of environmental selection pressure (81), thus selecting microbial taxa adapted to different conditions. In addition, the migration rate (m) is the same under low, medium, and high grazing intensities and is at a relatively low level, indicating that differences in grazing intensity are not sufficient to significantly affect the dispersal ability of wetland soil bacteria.

### Limitations of this study

This study provides new evidence for the analysis of the composition and assembly processes of soil bacterial communities in alpine wetlands under different grazing intensities. However, several limiting factors need to be considered (1). The cumulative effects and dynamic changes in long-term differences in the effects of grazing intensity on soil bacterial communities remain to be further investigated (2). Exploration of the dynamics of fungi and archaea under grazing and their interactions with bacteria is lacking (3). Although this study screened key bacterial taxa that respond to grazing disturbances from the perspectives of bacterial phyla and network hubs, there is a lack of discussion on the core functions of these taxa. These tasks will be carried out in subsequent research. (4) In this study, 0 cm–15 cm mixed soil samples were used. This sampling method tends to average out the characteristics of soil layers at different soil depths, potentially masking the vertical heterogeneity of the soil profile.

## Data Availability

Metagenomic sequencing data are fully available at CNCB under accession number CRA040156. Additional data are provided in the Supplemental Material.

## SUPPLEMENTAL MATERIAL

Supplemental tables spectrum.03791-25-s0001.docx Tables S1 to S4.

ASM does not own the copyrights to Supplemental Material that may be linked to, or accessed through, an article. The authors have granted ASM a non-exclusive, world-wide license to publish the Supplemental Material files. Please contact the corresponding author directly for reuse.
