# Supplementary material for: Bacterial diversity and community assembly mechanisms in alpine wetland soils under the influence of grazing
Source: Microbiol Spectr. 2026 Jul 6;14(8):e03791-25. doi: 10.1128/spectrum.03791-25 (PMC13436273; doi:10.1128/spectrum.03791-25)
Supplement: Supplementary file 1 [file spectrum.03791-25-s0001.docx]

**Supplementary Materials**

**Bacterial diversity and community assembly mechanisms in alpine wetland soils under the influence of grazing**

Xu Ai^1^, Cao Yu^1^, Ji Yangyue^1^, Zou Shuzhen^1,2^, Kang Di^1,2*^

1.Key Laboratory of Southwest China Wildlife Resources Conservation (Ministry of Education), China West Normal University, Nanchong, 637009, China

2.Key Laboratory of Environmental Science and Biodiversity Conservation (Sichuan Province), China West Normal University, Nanchong, 637009, China

* Corresponding author: Kang Di; E-mail address:kangyuyao@foxmail.com

**Table S1. Hub taxa under different grazing intensities**

|  | Phylum | L | M | H |
| --- | --- | --- | --- | --- |
| Degree hubs | Acidobacteriota | ASV43926, ASV70, ASV8548, ASV43937, ASV8482, ASV19268, ASV8698, ASV19139, ASV19160, ASV19190, ASV19140, ASV43917, ASV37665, ASV33563, ASV19254, ASV19143, ASV19137 | ASV269, ASV85, ASV4146, ASV8296, ASV30423, ASV8511, ASV30484, ASV24012, ASV20960  ASV20959, ASV55, ASV16848 | ASV36, ASV13640, ASV11866, ASV13610, ASV13553, ASV26334, ASV35947, ASV35946, ASV35955, ASV35950, ASV16334, ASV35968, ASV7529, ASV78, ASV20957 |
|  | Bacteroidota | ASV19176 | ASV21006 | - |
|  | Chloroflexota | ASV43928, ASV9176, ASV333 | ASV37, ASV20967, ASV21001 | ASV26245, ASV37 |
|  | Methylomirabilota | ASV43921, ASV43969, ASV43958 | - | ASV13548 |
|  | Pseudomonadota | ASV16904, ASV69, ASV8460, ASV16884, ASV19320, ASV8316 | ASV118, ASV3842, ASV720, ASV4053, ASV75, ASV8362, ASV24687 | - |
|  | RCP2-54 | - | ASV17253 | - |
| Closeness centraliy hubs | Acidobacteriota | ASV8599, ASV8404, ASV8691 | ASV12317, ASV8805, ASV8807 | ASV36, ASV13640, ASV11866, ASV13610, ASV13553, ASV26334, ASV35947, ASV35946, ASV35955, ASV35950, ASV16334, ASV35968 |
|  | Actinomycetota | ASV19169, ASV8512, ASV44013, ASV8287, ASV8947, ASV9021, ASV366 | ASV8542, ASV4036, ASV8411, ASV89, ASV8443, ASV12069 | ASV7529, ASV78, ASV20957 |
|  | Bacillota | ASV7613 | - | - |
|  | Pseudomonadota | ASV17085, ASV139 | ASV519, ASV21411, ASV3849 | - |
|  | Chloroflexota | - | ASV3897 | ASV26245, ASV37 |
|  | Methylomirabilota | - | - | ASV13548 |

**Table S2.Relationship between bacterial diversity and environmental factors**

|  | ALT | SLOP | ASP | CD | TN | SOM | TP | TK | AK | AP | pH | WC |
| --- | --- | --- | --- | --- | --- | --- | --- | --- | --- | --- | --- | --- |
| Chao | 0.17 | 0.117 | 0.333 | 0.099 | 0.267 | 0.255 | 0.312 | 0.047 | 0.313 | **0.643**** | -0.058 | -0.107 |
| Shannon | -0.196 | -0.061 | 0.125 | -0.302 | -0.059 | -0.037 | -0.008 | -0.126 | 0.206 | 0.366 | 0.357 | -0.377 |
| Simpson | -0.206 | 0.205 | -0.065 | 0.132 | -0.242 | -0.265 | -0.326 | -0.071 | -0.243 | -0.205 | 0.023 | -0.184 |

**Table S3.Relationship between key phylum and environmental factors**

|  | ALT | SLOP | ASP | CD | TN | SOM | TP | TK | AK | AP | pH | WC |
| --- | --- | --- | --- | --- | --- | --- | --- | --- | --- | --- | --- | --- |
| Pseud | **-0.728**** | -0.311 | -0.209 | **-0.574*** | **-0.730**** | **-0.724**** | **-0.638**** | 0.047 | -0.448 | -0.356 | **0.801**** | **-0.599**** |
| Acido | 0.368 | 0.192 | 0.325 | **0.495*** | 0.329 | 0.334 | 0.157 | -0.053 | 0.165 | -0.062 | **-0.476*** | 0.307 |
| Actin | 0.246 | **0.569*** | 0.152 | 0.111 | 0.193 | 0.189 | 0.082 | 0.127 | 0.131 | 0.187 | -0.108 | -0.018 |
| Chlor | -0.064 | -0.257 | -0.22 | 0.234 | -0.112 | -0.093 | -0.006 | -0.023 | -0.305 | 0.083 | -0.052 | 0.006 |
| Bacil | 0.387 | 0.112 | 0.396 | -0.139 | **0.563*** | **0.550*** | **0.649**** | 0.208 | **0.729**** | **0.534*** | -0.259 | 0.15 |
| Bacte | -0.465 | -0.189 | -0.094 | **-0.533*** | -0.463 | -0.43 | -0.322 | -0.124 | 0.102 | -0.337 | **0.560*** | -0.444 |
| Gemma | -0.13 | -0.259 | -0.386 | -0.109 | -0.198 | -0.167 | -0.082 | 0.248 | -0.307 | 0.035 | 0.103 | -0.158 |
| Methy | 0.391 | 0.016 | -0.214 | 0.279 | 0.445 | 0.416 | **0.493*** | -0.051 | -0.067 | 0.369 | **-0.486*** | **0.687**** |
| Verru | **0.710**** | 0.431 | 0.2 | 0.335 | **0.727**** | **0.739**** | **0.629**** | -0.141 | **0.633**** | 0.332 | **-0.650**** | **0.496*** |
| Lates | 0.246 | 0.195 | -0.084 | 0.353 | 0.254 | 0.222 | 0.321 | -0.29 | -0.016 | 0.077 | -0.438 | **.531*** |
| RCP2-54 | -0.017 | -0.27 | -.482* | 0.019 | 0.01 | -0.04 | 0.026 | -0.394 | -0.193 | 0.022 | -0.09 | 0.309 |
| NB1-j | **-0.825**** | -0.273 | -0.166 | **-0.675**** | **-0.774**** | **-0.786**** | **-0.633**** | -0.198 | -0.387 | -0.396 | **0.843**** | **-0.581*** |
| Bdello | 0.1 | -0.218 | -0.234 | 0.305 | 0.025 | -0.016 | 0.072 | -0.115 | -0.189 | 0.227 | -0.169 | 0.037 |
| Cyano | **-0.614**** | -0.415 | -0.418 | **-0.492*** | **-0.694**** | **-0.665**** | **-0.540*** | 0.227 | -0.441 | **-0.579*** | **0.648**** | -0.425 |
| Armati | **-0.626**** | -0.324 | -0.1 | -0.31 | **-0.571*** | **-0.555*** | -0.447 | -0.051 | -0.274 | -0.415 | **0.632**** | -0.327 |
| Entothe | -0.031 | -0.205 | 0.018 | 0.01 | -0.073 | -0.025 | -0.24 | 0.082 | 0.085 | -0.05 | 0.074 | -0.391 |
| Other | 0.014 | 0.094 | -0.124 | -0.141 | 0.169 | 0.104 | 0.161 | -0.412 | 0.052 | 0.002 | -0.13 | **0.489*** |

**Table S4. Data on environmental factors**

| NO. | Type | ALT  m | SLOP  degree | ASP  degree | CD | TN  g/kg | SOM  g/kg | TP  g/kg | TK  mg/kg | AK  mg/kg | AP  mg/kg | pH | WC  % |
| --- | --- | --- | --- | --- | --- | --- | --- | --- | --- | --- | --- | --- | --- |
| 1 | L | 3450.44 | 0 | 0 | 0.90 | 2.14 | 44.19 | 0.77 | 22.63 | 53.50 | 4.98 | 6.02 | 87.64 |
| 2 | L | 3447.65 | 8 | 55 | 0.80 | 2.90 | 55.48 | 0.80 | 19.42 | 108.50 | 11.45 | 6.25 | 104.14 |
| 3 | L | 3433.06 | 5 | 40 | 0.65 | 3.28 | 64.30 | 0.82 | 20.74 | 53.50 | 31.25 | 6.23 | 133.59 |
| 4 | L | 3429.18 | 9 | 180 | 0.85 | 2.27 | 49.26 | 0.74 | 19.76 | 109.50 | 10.08 | 6.21 | 60.78 |
| 5 | L | 3416.31 | 0 | 0 | 0.75 | 2.52 | 47.05 | 0.72 | 20.39 | 74.50 | 5.75 | 6.06 | 99.88 |
| 6 | L | 3425.02 | 0 | 0 | 0.80 | 3.19 | 64.47 | 0.82 | 19.25 | 79.50 | 13.82 | 5.87 | 154.33 |
| 7 | M | 3437.22 | 0 | 0 | 0.80 | 2.33 | 51.94 | 0.73 | 21.17 | 77.50 | 7.05 | 6.37 | 103.65 |
| 8 | M | 3434.09 | 0 | 0 | 0.70 | 2.78 | 60.9 | 0.69 | 19.40 | 84.50 | 6.68 | 6.10 | 81.91 |
| 9 | M | 3417.21 | 0 | 0 | 1.00 | 2.05 | 38.63 | 0.70 | 20.44 | 41.50 | 17.25 | 6.13 | 65.19 |
| 10 | M | 3420.71 | 10 | 30 | 0.80 | 2.24 | 44.81 | 0.56 | 20.82 | 41.50 | 12.12 | 5.68 | 55.64 |
| 11 | M | 3517.29 | 5 | 160 | 0.65 | 4.23 | 89.42 | 0.90 | 21.14 | 176.50 | 31.25 | 6.37 | 50.79 |
| 12 | M | 3442.5 | 0 | 0 | 1.00 | 2.22 | 46.73 | 0.62 | 19.47 | 45.50 | 5.75 | 6.63 | 59.76 |
| 13 | H | 3226.42 | 0 | 0 | 0.30 | 0.73 | 13.09 | 0.63 | 19.07 | 89.50 | 3.05 | 8.46 | 40.65 |
| 14 | H | 3225.29 | 0 | 0 | 0.75 | 0.77 | 16.46 | 0.60 | 19.05 | 49.50 | 4.72 | 8.36 | 38.92 |
| 15 | H | 3224.33 | 0 | 0 | 0.40 | 0.46 | 9.64 | 0.58 | 22.41 | 27.50 | 4.28 | 8.68 | 43.76 |
| 16 | H | 3224.62 | 0 | 0 | 0.50 | 0.57 | 12.45 | 0.56 | 20.04 | 36.50 | 4.65 | 8.79 | 35.63 |
| 17 | H | 3266.72 | 0 | 0 | 0.50 | 0.96 | 20.65 | 0.64 | 20.37 | 32.50 | 7.78 | 8.03 | 57.34 |
| 18 | H | 3262.88 | 2 | 225 | 0.50 | 1.34 | 23.82 | 0.62 | 21.51 | 53.50 | 8.82 | 8.10 | 69.60 |

Note: Laboratory instrument readings were reported to three decimal places for available phosphorus (AP) and one decimal place for available potassium (AK). In the laboratory calculation, AP was calculated as AP = (reading − 0.0055 [batch blank correction]) × 50 [extraction volume, mL] / 2.50 [sample mass, g] × 25/15 [color-development volume/aliquot volume] = (reading − 0.0055) × 100/3. AK was calculated as AK = (reading − 0.15 [batch blank correction]) × 20 [extraction/final solution volume, mL] / 2.00 [sample mass, g] = 10 × reading − 1.5. Given these reporting precisions and calculation factors, values reported to two decimal places show repeated terminal decimal patterns, with AP commonly ending in 2, 5, or 8 and AK having a fixed 0.5 decimal fraction before final formatting.
